# Chest computed tomography to evaluate lymphocytic interstitial pneumonia

**DOI:** 10.1590/0100-3984.2020.53.5e1

**Published:** 2020

**Authors:** Miriam Menna Barreto, Rosana Souza Rodrigues

**Affiliations:** 1 Universidade Federal do Rio de Janeiro (UFRJ), Rio de Janeiro, RJ, Brazil.

Lymphocytic interstitial pneumonia (LIP) is a benign lymphoproliferative disease characterized by infiltration of the pulmonary interstitium with lymphoid tissue^(1)^. It is usually associated with various conditions, most commonly autoimmune diseases, such as Sjögren’s syndrome, rheumatoid arthritis, systemic lupus erythematosus, or immunodeficiency states, such as HIV infection^(2)^. However, rarely LIP is idiopathic and, in those cases is classified in the group of rare idiopathic interstitial pneumonias according to the latest update from the American Thoracic Society/European Respiratory Society^(3)^.

Chest computed tomography (CT) is considered to be the gold standard to evaluate diffuse pulmonary diseases, being a fundamental tool to characterize interstitial pneumonias, including LIP. The most common LIP tomographic characteristics are pulmonary air cysts, generally presenting in a few number, diffusely distributed in the lungs, and often associated with ground-glass opacities^(4)^. CT has good sensitivity to detect and characterize these cysts (distribution, size, extension, and wall regularity) and to identify other associated findings^(5)^.

Although the differential diagnosis of diffuse cystic diseases of the lungs is wide, most of these conditions are rare. Lymphangioleiomyomatosis and Langerhans cell histiocytosis are the diseases that mostly present with pulmonary air cysts. Other rare conditions, such as Birt-Hogg-Dubé syndrome, follicular bronchiolitis, and light-chain deposition disease can progress to multiple air cysts, being included in the differential diagnosis of LIP^(6)^. A major diagnostic challenge is the presence of pulmonary air cysts in patients with acquired immunodeficiency syndrome. In these patients, this finding may be due to pneumocystosis or LIP. The recognition of the tomographic characteristics of lung cystic diseases is essential to narrow the differential diagnosis.

The article by Louza et al.^(7)^, published in this issue of Radiologia Brasileira, reported the main chest CT findings of 36 patients diagnosed with LIP, 13 of whom were confirmed by a lung biopsy while the others by clinical, laboratory, and radiological criteria. The study has a robust sample considering the disease rarity and data in the literature. The immunological diseases mostly associated with LIP are Sjögren’s syndrome and HIV infection^(8)^, corroborating the study by Louza et al.^(7)^, in which the authors found LIP to be associated with Sjögren’s syndrome in most cases (42%). In addition to LIP, Sjögren’s syndrome may be associated with organizing pneumonia and usual interstitial pneumonia, and in those cases may require a transbronchial or surgical lung biopsy^(9)^.

The study by Louza et al. is valuable, mainly because recognizing the tomographic characteristics of each disease pattern is fundamental for the correct interpretation of radiological findings, aimed at narrowing the differential diagnosis and avoiding invasive procedures. In that study, the most common LIP CT scan findings were multiple, rounded, thin-walled air cysts mostly measuring less than 20 mm, with diffuse distribution through the lungs, associated with ground-glass opacities or small nodules. The authors also highlighted the high prevalence of LIP associated with amyloidosis, with the concomitant finding of air cysts and nodules on CT being suggestive of this association, particularly when the nodules are calcified, which has not been previously described in LIP studies with relevant samples^(4)^.

In conclusion, the study by Louza et al.^(7)^ shows detailed tomographic characteristics that help the radiologist suspect LIP, being useful in the differential diagnosis of other lung diseases presenting with air cysts, and also raise suspicion of associated clinical conditions, mainly immunodeficiency and autoimmune diseases.
